# Early-life serum protein profiles associated with bronchopulmonary dysplasia in extremely preterm infants

**DOI:** 10.1186/s12931-026-03920-y

**Published:** 2026-09-18

**Authors:** Susanna Klevebro, Mohit B. Panwar, Dirk Wackernagel, Ingrid Hansen Pupp, Sophia Björkander, Hanna Danielsson, Mathias Uhlén, Liv Vallin, Karin Sävman, Aldina Pivodic, Ann Hellström, Anders K. Nilsson

**Affiliations:** 1 Department of Clinical Science and Education Södersjukhuset, Karolinska Institutet, Stockholm, Sweden; 2 Sachs’ Children and Youth Hospital, Stockholm, Sweden; 3 The Sahlgrenska Centre for Pediatric Ophthalmology Research, Department of Clinical Neuroscience, Institute of Neuroscience and Physiology, Sahlgrenska Academy, University of Gothenburg, Gothenburg, Sweden; 4 Division of Neonatology, Department of Pediatrics, University Medical Center of the Johannes Gutenberg-University Mainz, Mainz, Germany; 5 Department of Clinical Sciences, Lund, Pediatrics, Lund University, Lund, Sweden; 6 Skåne University Hospital, Lund, Sweden; 7 Department of Women’s and Children’s Health Karolinska Institutet, Stockholm, Sweden; 8 Science for Life Laboratory, Department of Protein Science, KTH-Royal Institute of Technology and Department of Neuroscience, Karolinska Institutet, Stockholm, Sweden; 9 Department of Pediatrics, Institute of Clinical Sciences, Sahlgrenska Academy, University of Gothenburg, Gothenburg, Sweden; 10 Deptartment of Neonatology, The Queen Silvia Children’s Hospital, Sahlgrenska University Hospital, Region Västra Götaland, Gothenburg, Sweden; 11 Department of Ophthalmology, Sahlgrenska University Hospital, Region Västra Götaland, Gothenburg, Sweden

**Keywords:** Preterm infant, Lung development, Bronchopulmonary dysplasia, Proteomics, Biomarker

## Abstract

**Background:**

Bronchopulmonary dysplasia (BPD) affects the most immature preterm infants and can result in long term pulmonary morbidity. Yet, how prenatal and neonatal exposures perturb early proteome maturation and contribute to BPD pathogenesis remains unclear.

**Methods:**

In a cohort of 176 extremely preterm infants (born < 28 weeks gestational age) previously enrolled in a multicenter randomized trial, 825 longitudinal serum samples were collected the first five postnatal weeks. 98 of 176 infants were defined as having BPD based on need for any respiratory support at 36 weeks postnatal age. Proteomic analysis was conducted using proximity extension assay from Olink. Mixed models, adjusted for gestational age, study center, and randomization group, also including an interaction term between BPD and time, were used to analyse differences in longitudinal protein trajectories by BPD status. False discovery rate correction was applied.

**Results:**

Out of 538 analysed proteins, 108 exhibited distinct longitudinal trajectories in infants with BPD. Among the top significant proteins, several have previously been associated with BPD (e.g. lower Secretoglobin family 3A member 1 [SCGB3A1] and higher intercellular adhesion molecule 1 [ICAM-1]), while others represent novel findings (e.g. lower Leukocyte differentiation antigen CD84). Network and functional enrichment analysis revealed involvement in multiple biological processes, with many of the top significant proteins related to immune function. Some proteins showed associations specific to more mature infants (e.g. Stem cell factor).

**Conclusion:**

Differences in early serum protein trajectories are evident in infants who develop BPD. Our findings suggest associations consistent with proposed pathophysiological mechanisms. Clinical factors likely interact with immune-related proteins in the development of BPD. Mitigating inflammation by stabilising potential protective factors could be important for BPD prevention.

**Trial registration:**

ClinicalTrial.gov identifier NCT03201588.

**Supplementary Information:**

The online version contains supplementary material available at https://doi.org/10.1186/s12931-026-03920-y.

## Introduction

Lung development begins in-utero and preterm birth disrupts this process, increasing the risk of chronic lung disease [1]. Bronchopulmonary dysplasia (BPD) is an indicator of neonatal lung morbidity traditionally defined based on the need for supplementary oxygen or respiratory support at 36 weeks postmenstrual age (PMA) [2]. Recent studies have found that the mode of respiratory support is more strongly associated with long term outcomes [3].

The pathophysiological mechanisms behind BPD are not fully known. Several processes are likely to contribute, including cellular injury and disturbed repair, mitochondrial dysfunction and extracellular matrix remodelling [1]. Dysregulated immune modulation is likely important for the development of preterm associated lung disease [4], and airway findings in adult survivors of preterm birth suggest persistent inflammatory processes and disturbed immune modulation [5].

Gestational age (GA) at birth is the most prominent risk factor for disturbed lung development [1]. The risk of BPD is inversely associated with GA with a prevalence ranging from 70% in infants born before 25 weeks GA to 41% in infants born in 25 to 27 weeks of GA [6]. Out of perinatal exposures, foetal growth restriction manifesting as being born small for GA, and factors related to inflammatory processes such as a foetal inflammatory response, and early sepsis have been associated with increased risk of BPD and chronic lung disease [7, 8].

The diagnosis of BPD results in heterogenous lung disease later in life with differences in radiology, histology and treatment response [1]. The existence of multiple BPD phenotypes may reflect distinct underlying mechanisms of disease development. A better understanding of how clinical characteristics relate to molecular pathways would increase the possibilities of targeted preventions.

The aim of this study was to elucidate pathophysiological mechanisms of prematurity-associated lung disease by identifying biomarkers associated with increased risk of BPD, potentially specific in combination with clinical characteristics. This was done utilizing a cohort of extremely preterm infants from a randomized trial with frequent longitudinal proteomic measurements during the first postnatal weeks [9].

## Methods

### Study population

The Mega Donna Mega trial (ClinicalTrial.gov identifier NCT03201588) was a multicenter study of extremely preterm infants randomized to receive enteral supplementation with the polyunsaturated fatty acids arachidonic acid (AA) and docosahexaenoic acid (DHA) from birth to 40 weeks PMA, or standard care. The primary objective was prevention of retinopathy of prematurity (ROP), with BPD evaluated as a secondary outcome [10]. AA:DHA supplementation reduced the risk of severe ROP [10] but was not associated with BPD and not with protein trajectories the first postnatal weeks, as reported previously [11, 12].

### Clinical variables and definition of outcome

Outcome was BPD defined according to the Jensen et al. classification, based on the mode of respiratory support at 36 weeks PMA [3]. The analysis compared infants with no BPD to infants with BPD, defined as any requirement for respiratory support at 36 weeks PMA. For a subset of proteins, trajectories were also visualized by BPD grade, classified as follows: Grade 1, low-flow nasal cannula (maximum flow < 2 L/min); Grade 2, high-flow nasal cannula (≥ 2 L/min), continuous positive airway pressure, or non-invasive positive-pressure ventilation; and Grade 3, invasive positive-pressure ventilation.

Birth weight (BW) z-scores were calculated based on equations suggested by Fenton and Kim [13]. Clinical variables were prospectively collected during the study and has been further validated. Detailed clinical data was available for the first four postnatal weeks. Sepsis was defined as positive blood culture unless the blood culture indicated Coagulase-negative staphylococci, in which case an elevated C-reactive protein (CRP) > 20 mg/L was required. From daily records of administered medications, the running cumulative dose of given corticosteroids per last known weight of the infants was calculated. Infants received both betamethasone and hydrocortisone. Betamethasone dosages were converted into hydrocortisone equivalents (1:35). Enteral and parentereral nutrition regimes have been previously published [10].

### Sampling and proteomics analysis

Infants were sampled at approximately postnatal day 1, 3, 7, 14, and 28, and then approximately every other week until discharge. This study includes proteomic data from the first five postnatal weeks, the period with most clinical and sampling data, and a period likely to be of importance for BPD development.

In total, 538 proteins from six proteomic panels (Cardiometabolic v.3603, Cardiovascular II v.5006, Cardiovascular III v.6113, Development v.3512, Metabolism v.3402, and Inflammation v.3022) were measured using the proximity extension assay (PEA) by Olink Proteomics (Uppsala, Sweden) [14]. Protein levels are expressed in normalized protein expression (NPX), a relative unit on a log2 scale. A high value represents higher serum levels.

### Statistics

Continuous variables are described by median and range, categorical variables by counts and percentages. Differences in clinical characteristics between infants with and without BPD were analysed using logistic regression, unadjusted and adjusted for GA.

Differences in protein trajectories during the first five postnatal weeks were analysed using linear mixed-effects models, in which postnatal age was modelled using natural cubic splines with three degrees of freedom. Separate models were fitted for each of the 538 proteins. The models included BPD status (yes/no), randomization group in the original trial (AA:DHA supplementation or standard care), study center, and GA as fixed effects, with random intercepts for subjects. Linear mixed-effects models inherently account for missing data through maximum likelihood estimation under a missing-at-random assumption, and no imputation was performed. A likelihood ratio test compared a reduced model without interaction terms to a model including a BPD × postnatal age interaction to determine whether longitudinal protein trajectories overall differed between infants with and without BPD. False discovery rate (FDR) adjustment to control for multiple testing was based on Benjamini–Hochberg correction and q-value < 0.05.

Proteins with overall FDR significant longitudinal trajectories, based on a likelihood ratio test, were selected for network analysis. Selected proteins were mapped to a Protein–Protein Interaction (PPI) network using STRINGdb v12.0 for Homo sapiens. A STRING subnetwork was retrieved using the default interaction score threshold. Community detection was performed using the Louvain algorithm implemented in igraph with the resolution parameter set to 2.5. Functional enrichment analysis was performed separately for each cluster using gprofiler2 with FDR correction using the Benjamini–Hochberg procedure. Enrichment was assessed for Gene Ontology biological process, molecular function, and cellular component, KEGG pathways, and WikiPathways.

Proteins with an FDR significant interaction term between BPD and postnatal age in any of the spline functions in the adjusted mixed-effect model were selected for further analyses of associations with clinical variables. To study explained variance in protein area under the curve, calculated using the trapezoid method, variance partitioning was performed using the variancePartition package, which estimates the proportion of outcome variance attributable to individual predictors within a multivariable linear modeling framework [15]. Models included GA, study center, randomization group, BW z-score, sex, surfactant treatment, sepsis, days of mechanical ventilation, days of parenteral nutrition, cumulative steroid dose, and cumulative blood transfusions during the first four postnatal weeks. To explore differences in BPD-related protein trajectories in subgroups, the highest and lowest tertiles of GA were compared with the median tertile and the lowest BW z-score tertile was compared with the combined median and highest tertile. Visual inspection of the protein trajectories by BPD in the subgroups were performed and effect modification by subgroup was tested by introducing a BPD × subgroup interaction term in the mixed effect model.

All tests were two-tailed. All analyses were performed by using R version 4.5.0.

## Results

### Clinical characteristics

**Fig. 1:**
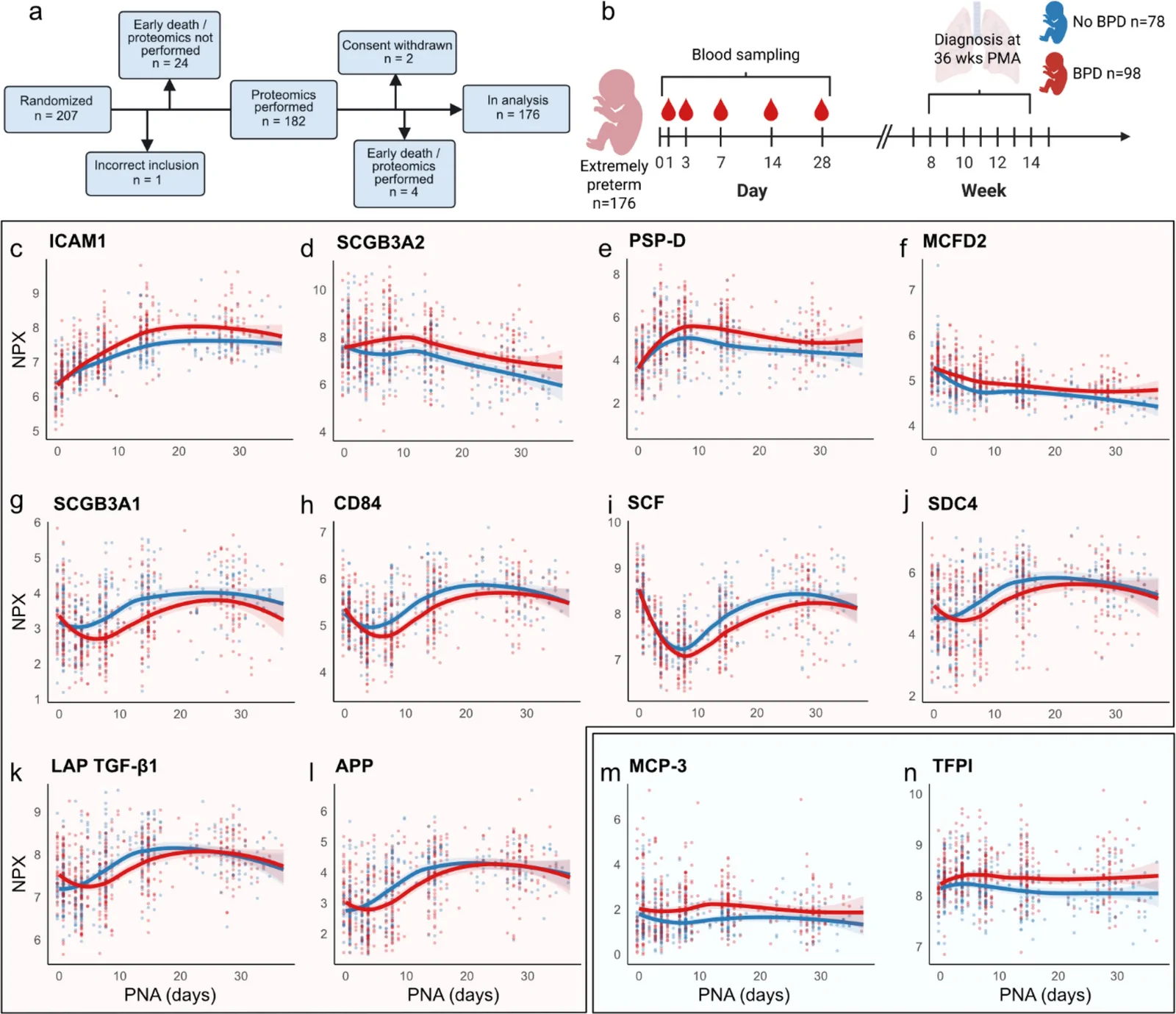
**a** Flow-chart. **b** Study overview. **c**-**l** Longitudinal protein trajectory by BPD outcome in selected proteins with FDR significant interaction between BPD and postnatal age. **m**–**n** Proteins with FDR significant main effect of BPD in the mixed model. Points represent individual measurements and lines indicate LOESS smooths with shaded areas denoting 95% confidence intervals. PNA, postnatal age; NPX, normalized protein expression

Out of 207 infants randomized in the original trial, one was incorrectly included, two withdrew consent, and 28 died before 36 weeks PMA (Fig. 1a). In total, 176 infants had information regarding BPD grade at 36 weeks PMA. Of the included infants, 78 infants (44%) did not have BPD, and 98 infants (56%) had any grade of BPD (Fig. 1b). Three (2%) infants had BPD grade 3, 50 (28%) infants had BPD grade 2, and 45 (26%) infants had BPD grade 1. There was no difference in the prevalence of BPD in infants who had received AA:DHA supplementation 49/82 (60%) or standard care 49/94 (52%). Mean number of serum samples for proteomics per infant was 4.7, without differences between the BPD groups.

Compared to infants without BPD, infants with BPD had lower GA, received a higher cumulative dose of postnatal steroids, more blood transfusions, and had more days with mechanical ventilation in the first four postnatal weeks. There were no significant differences in sex, or BW z-score. Differences between groups in BW, received surfactant, number of days with parenteral nutrition, minimum value of platelet count (Plt), minimum value of Hb, and maximum value of CRP in the first four postnatal weeks did not persist after adjustment for GA (Table 1). Prenatal and neonatal characteristics in BPD groups per GA tertile are shown in Table S1 in Additional file 1. In the highest GA tertile, *i.e.* the most mature infants, those with BPD more often had sepsis 6/22 (27%) in the first four postnatal weeks compared to those without BPD 2/33 (6%) (OR 5.8, 95% CI 1.1–32; *p* = 0.044), although the absolute numbers were low.

**Table 1:** Prenatal and neonatal characteristics in infants without BPD and with any grade of BPD

Variables	No BPD *N* = 78	BPD any grade *N* = 98	*P*-value
Gestational age (weeks)	26.4 (23.0; 27.9)	25.1 (23.0; 27.9)	< 0.001
Birth weight (g)	868 (470; 1330)	712 (425; 1345)	< 0.001
Standardized birth weight (z-score)	0.2 (−2.0; 1.8)	0.2 (−2.3; 2.3)	0.16
Sex (Female)	34 (44%)	41 (42%)	0.82
Surfactant	47 (60%)	74 (76%)	0.03
Ventilator days, 4 weeks	0 (0; 28)	18 (0; 28)	< 0.001^*^
Cumulative dose postnatal steroids, 4 weeks (mg/kg)	0 (0; 28)	5 (0; 201)	< 0.001^*^
Sepsis, 4 weeks	8 (10%)	15 (15%)	0.33
Sum blood transfusion, 4 weeks (mL/kg)	26 (0; 139)	60 (0; 147)	< 0.001^*^
Days with parenteral nutrition, 4 weeks	12 (4; 28)	16.5 (4; 28)	0.003
Min Hb, 4 weeks (g/L)	104 (40; 154)	101 (38; 149)	0.005
Min Plt, 4 weeks (g/L)	164.5 (36; 400)	134.5 (8; 364)	0.004
Max CRP, 4 weeks (mg/L)	4.6 (0.3; 142)	11.5 (0.3; 217)	0.04

Data are presented as median (minimum, maximum), number of observations (percentage)

*BPD* bronchopulmonary dysplasia, *CRP* C-reactive protein, *Hb* Hemoglobin, *Plt* Platelet count

^*^P-value < 0.05 also in the analysis adjusted for gestational age

### Differences in protein trajectories in the neonatal period depending on BPD outcome

The longitudinal protein trajectory during the first five postnatal weeks differed in infants with any grade of BPD compared to those with no BPD in 108 (20%) of the analysed proteins (Table S2, Additional file 2). Of the 108 proteins, 35 proteins had an FDR significant interaction term between BPD and postnatal age in any of the spline functions in the adjusted mixed model (Table S3, Additional file 2). The longitudinal trajectories varied among the proteins: some proteins showed higher levels in infants with BPD, (e.g. intercellular adhesion molecule 1 [ICAM-1], secretoglobin family 3A member 2 [SCGB3A2], pulmonary surfactant-associated protein D [PSP-D], and multiple coagulation factor deficiency protein 2 [MCFD2] Fig. 1c-f), whereas other proteins were lower in infants with BPD (e.g. secretoglobin family 3A member 1 [SCGB3A1], leukocyte differentiation antigen CD84 [CD84], stem cell factor [SCF], and syndecan-4 [SDC4] Fig. 1g-j). Of the proteins with lower levels in infants with BPD, the BPD group had a steeper decline in some proteins (e.g. SCGB3A1 and CD84), and a later increase in others (e.g. latency-associated peptide transforming growth factor beta-1 [LAP TGF-β1] and amyloid precursor protein [APP] Fig. 1k-l) compared to the group without BPD. Longitudinal protein trajectories by graded BPD outcome for the 35 proteins with an FDR-significant interaction term between BPD and postnatal age are visualized in Figure S1, Additional file 1.

Two proteins (monocyte chemoattractant protein-3 [MCP-3] and tissue factor pathway inhibitor [TFPI]) did not show longitudinal differences between BPD groups (no FDR significant BPD × time interaction) but an FDR significant main effect of BPD in the mixed model, showing consistently higher levels in infants with BPD (Fig. 1m-n).

### Network and functional enrichment analysis

**Fig. 2:**
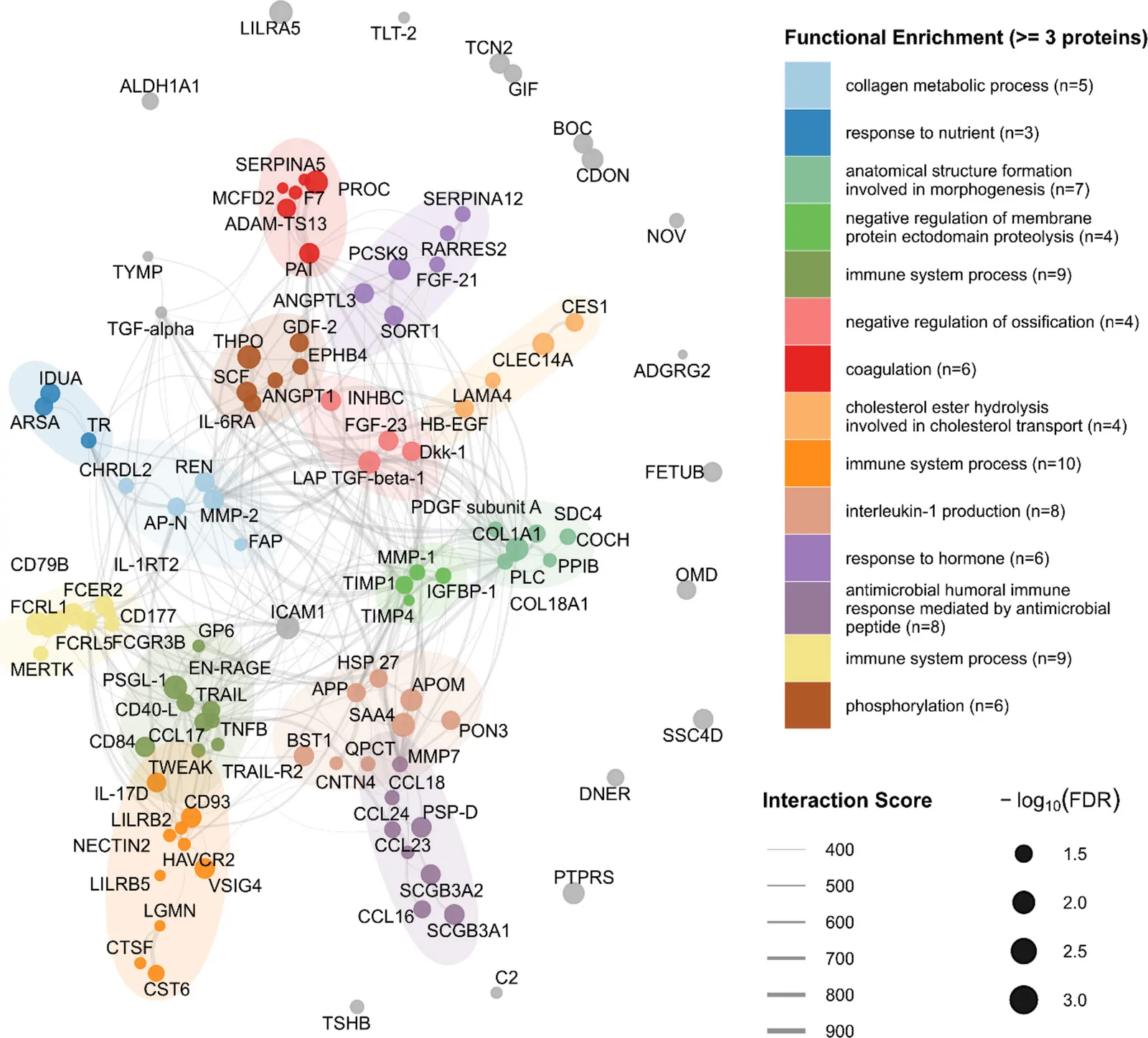
Network of 108 proteins with FDR significant differences in longitudinal trajectory associated with BPD. Illustrated with interaction scores from STRINGdb and functional enrichment using gprofiler2, details and protein abbreviations are shown in Table S2, Additional file 2

In the network analysis, based on the 108 proteins with overall FDR significant longitudinal protein trajectories, most proteins showed associations with other quantified proteins, forming interconnected clusters. We identified distinct “hubs” of proteins with functions previously implicated in the pathophysiology of BPD, including immune functions and inflammation (e.g., immune system process activation/regulation, response to cytokine, interleukin-1 [IL-1] production), angiogenesis, and tissue remodelling (e.g., anatomical structure formation, programmed cell death, collagen catabolic process). Additionally, we observed proteins involved in biological processes less commonly linked to BPD development, such as response to hormone, and coagulation (Fig. 2).

The 35 proteins selected for further analyses based on an FDR significant BPD × time interaction term in the main model were grouped into functional hubs spanning a wide range of biological processes. Notably, 16 of the proteins clustered within immune-related hubs (response to chemokine, IL-1 production, immune system activation, and regulation).

### Protein variability explained by clinical variables

**Fig. 3:**
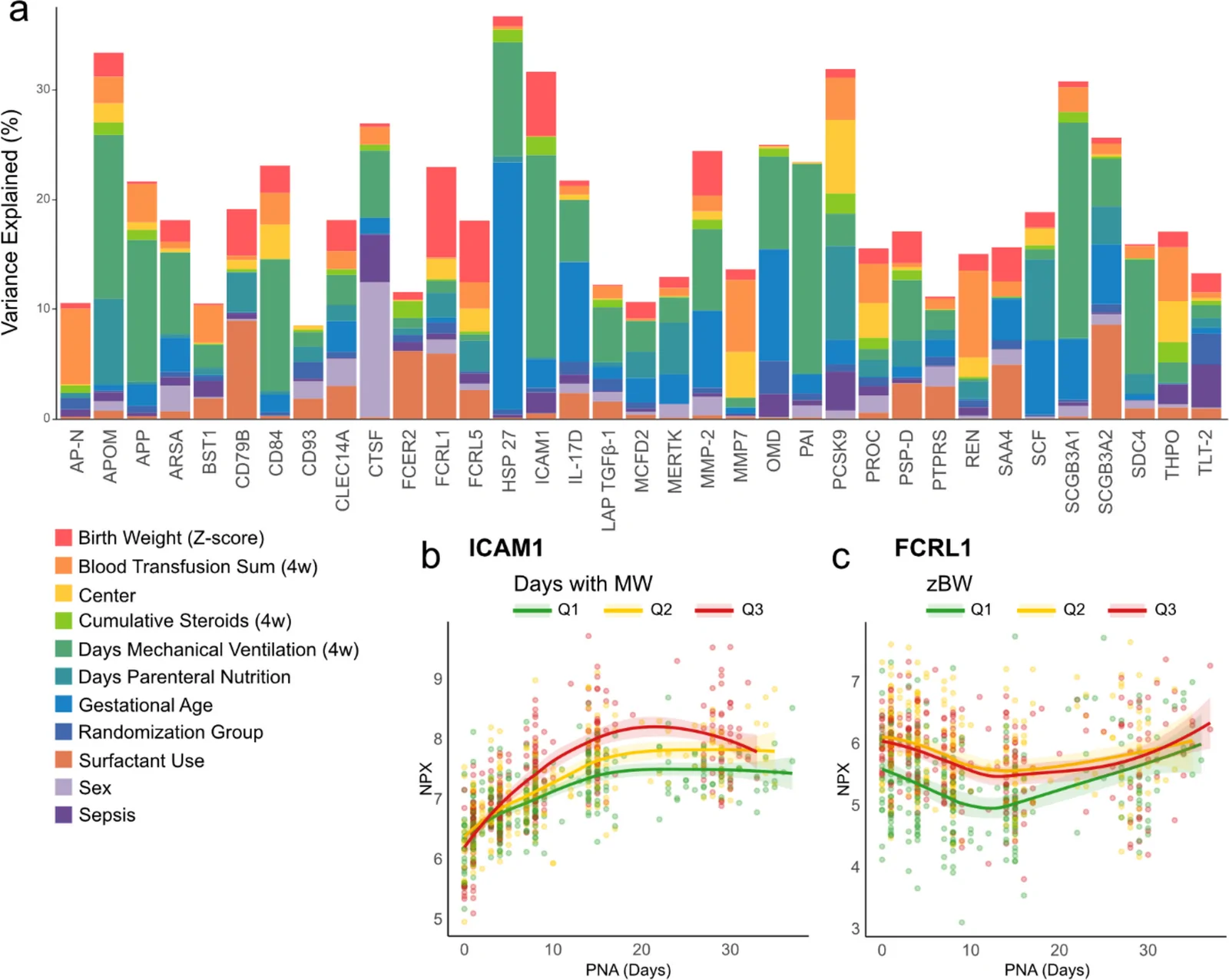
Clinical variables and variability in protein levels. **a** Proportion of variability explained by clinical variables. Longitudinal protein levels in **b** ICAM-1 by tertiles of ventilator days, and **c** FCRL1 by tertiles of BW z-score

Among the 35 proteins with significant BPD × time interaction, variability in protein levels during the first four postnatal weeks was only partly explained by clinical factors. The 12 selected clinical variables (GA, study center, randomization group, BW z-score, sex, use of surfactant, sepsis, days of mechanical ventilation, days of parenteral nutrition, cumulative postnatal steroid dose, and total blood transfused the first four postnatal weeks) explained 9–37% of the variability in individual protein levels during the first four postnatal weeks (Fig. 3a). Clinical variables and variability in protein levels for all 108 proteins are shown Figure S2 in Additional file 1. For several of the proteins (e.g. SCGB3A1, PAI, ICAM-1, and APOM), ventilator days was associated with the largest proportion of variability. ICAM-1 levels increased in infants with more ventilator days (Fig. 3b). GA specifically influenced the variability of heat shock protein 27 (HSP27). Fc receptor-like protein 1 [FCRL1] was one of the proteins with variance influenced by BW z-score, showing lower levels in the lowest z-score tertile (Fig. 3c).

### Early postnatal protein trajectory and BPD in relation to gestational age and growth restriction

GA and BW z-score are perinatal factors associated with BPD that were also found to explain protein variability; therefore, we explored if the association between BPD and postnatal protein trajectory differed by GA or BW z-score tertiles among the 35 proteins selected for further analysis.

**Fig. 4:**
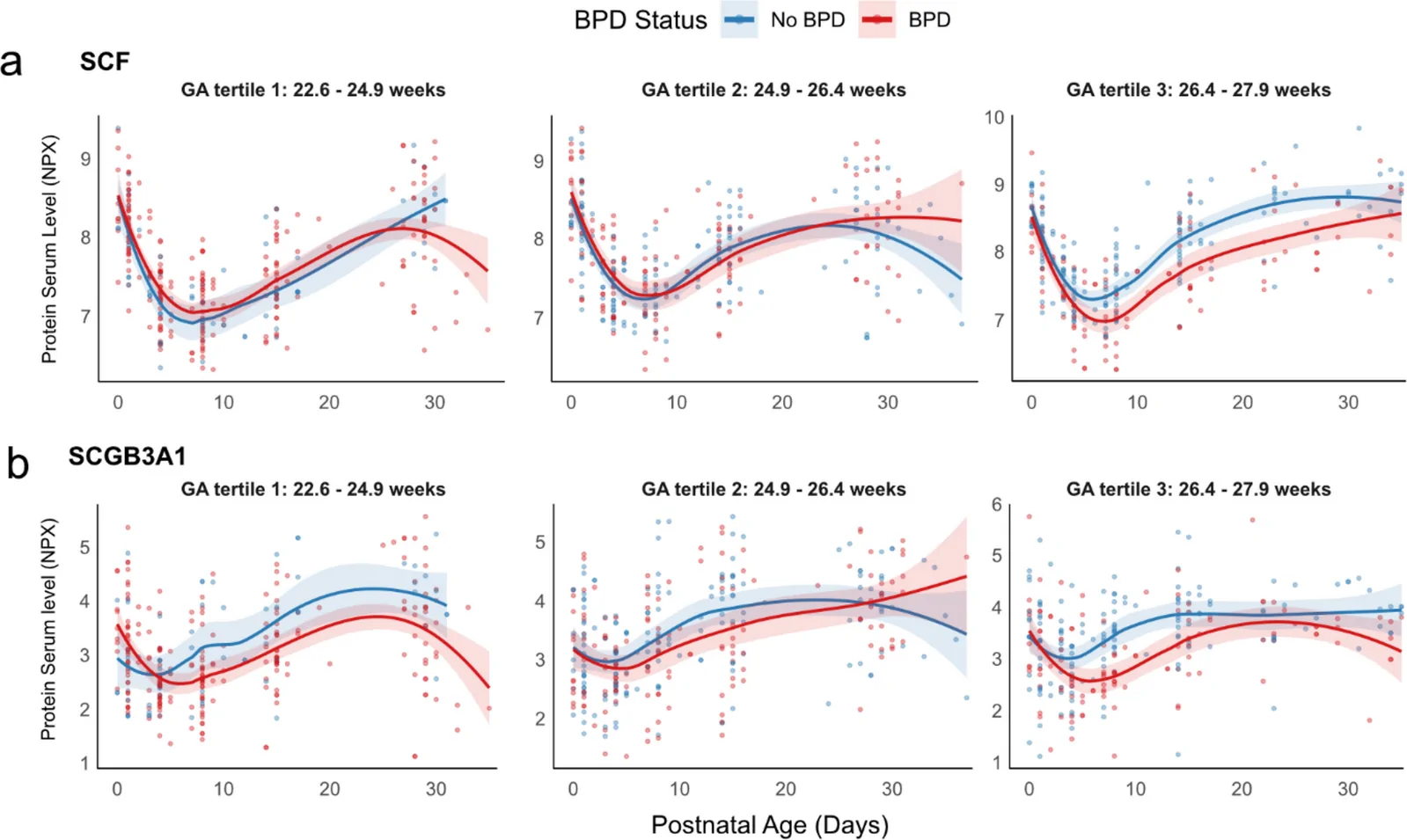
Longitudinal protein trajectory by BPD stratified by GA tertiles showing the most immature infants in tertile 1 (left panel) and the most mature infants in tertile 3 (right panel) for **a** SCF and **b** SCGB3A1

Six proteins differed in the association with BPD between GA tertiles, (nominal *p*-value for interaction < 0.05). For SCF, only the most mature infants showed protein level differences depending on BPD outcome (Fig. 4a). For SCGB3A1, infants with BPD showed an early deviation in protein levels, in GA tertile 3, with the most mature infants, and less obvious separation in the lower GA tertiles (Fig. 4b). For osteomodulin (OMD), IL17D, HSP27, and receptor-type tyrosine-protein phosphatase S (PTPRS) the pattern was less clear (Figure S3 in Additional file 1). Range and clinical variables per GA tertile are shown in Table S1 in Additional file 1.

When comparing the lowest BW z-score tertile (median −0.6, min −2.3; max −0.2) to the two higher tertiles (median 0.5, min −0.1; max 2.3), the nominal *p*-value for interaction was < 0.05 for CD84 although the pattern was similar in both groups (Figure S4a in Additional file 1). The proteins LAP TGF-β1and APP had longer duration of lower levels in infants with BPD in the lowest BW z-score tertile at visual inspection of protein trajectory over time, but the nominal *p*-value for interaction was 0.05 and 0.12 respectively (Figure S4b-c in Additional file 1).

## Discussion

This study shows overall differences in protein trajectories during the first five postnatal weeks in infants with BPD when compared to infants without BPD. Several of the identified proteins have previously been linked to BPD development (e.g. ICAM-1 and the secretoglobins SCGB3A1 and SCGB3A2), whereas others, such as CD84, bone marrow stromal cell antigen 1 (BST-1), MCFD2, SDC4, and proprotein convertase subtilisin kexin 9 (PCSK9), have not been reported in relation to BPD before. The proteins associated with BPD represent a wide range of functions, illustrating the complex pathophysiological mechanisms underlying BPD development. Notably, several of the top proteins identified in our analysis are involved in immune-related processes.

Similar to our results, a recent study of acute respiratory distress syndrome in children identified ICAM-1 as a central hub in the network analysis [16]. Multiple studies have shown increased postnatal levels of ICAM-1 in infants who later develop BPD [17, 18]. ICAM-1 is a cell-surface glycoprotein expressed on endothelial cells, epithelial cells, and immune cells. It facilitates migration of leukocytes and stimulates cell-to-cell contact that drive production of proinflammatory cytokines. Elevated levels of ICAM-1 have been associated with sepsis in preterm and term infants [19, 20]. BST-1, found to be elevated in BPD infants around the third postnatal week in our study, is another cell-surface protein that acts as an immune regulator. Increased expression of BST-1 has been shown in children with sepsis [21] but has to our knowledge not previously been associated with BPD.

Proteins of the secretoglobin family are involved in immune regulatory and anti-inflammatory processes and are thought to have functions in barrier tissues [22]. The most well-described member of the secretoglobin family, SCGB1A1, seems to have a protective role in airway diseases. Low levels have been related to neonatal lung disease as well as prematurity related lung disease later in life [23, 24]. SCGB1A1 was not measured in this study. However, we found the secretoglobin family members SCGB3A1 and SCGB3A2 to be associated with BPD, showing opposing longitudinal protein patterns in the BPD groups. Both SCGB3A1 and SCGB3A2 have been associated with BPD in mice and in tissue from deceased infants with BPD [25].

In our study, the levels of PSP-D, a surfactant protein also involved in microbial defence, was higher in infants developing BPD. Previous studies have yielded conflicting results regarding PSP-D and BPD [26, 27]. De Luca et al. showed lower early levels of PSP-D in the airways of infants who develop BPD and also increasing levels with increasing GA [26]. Vinod et al. could not demonstrate any correlation between PSP-D serum levels in the first postnatal week and GA, BW, or BPD [27]. De Luca et al. also reported higher levels of PSP-D in the airways of infants exposed to foetal inflammation or chorioamnionitis [26], and higher levels have been reported in preterm infants with sepsis [28]. PSP-D is a biomarker of chronic obstructive lung disease showing higher levels in subjects with disease and increased levels preceding exacerbations but suppressed levels relating to oral corticosteroid treatment [29].

PSP-D, the secretoglobins, and ICAM-1 are involved in immune regulation and can be activated via multiple pathways, including endothelial or epithelial stimuli. Exposures affecting these pathways may also include treatments of impaired pulmonary function, such as mechanical ventilation and corticosteroids. Given the observational design of this study, it is not possible to determine whether the observed associations reflect underlying vulnerability, exposures, disease severity, or BPD-specific pathophysiology.

Another “hub” of proteins—LAP TGF-β1, plasminogen activator inhibitor-1 [PAI], APP, CD84, and SDC4—is involved not only in immune regulation but also in growth and vascular development. These proteins had postnatal protein trajectories that differed in infants who developed BPD in this study. It has been previously shown in this cohort that the levels of these proteins were negatively associated with thrombocytopenia the first four postnatal weeks [30]. Granules from platelets are known to carry proteins involved in inflammation, immune response, angiogenesis, and atherosclerosis [31]. TGF-β1is a multifunctional cytokine involved in cell differentiation and proliferation as well as immune regulation. TGF-β1has functions in the coordination of alveolar vascular development [32], and the TGF-β1 pathway has been implicated in BPD pathophysiology [33]. TSP-1 is an angiogenetic protein that activates TGF-β1. TSP-1 is produced by platelets and lung cells, and there is an increased expression in platelets of ventilated preterm infants [34]. Growth restriction has been associated with decreased TGF-β1expression and additional downregulation of PAI affecting extracellular matrix remodelling in animal models [35]. Cederquist et al. showed that infants who developed BPD had higher levels of PAI in the airways during the first postnatal week [36]. Other studies have also shown an upregulation of PAI, both in experimental studies of senescensed lung fibroblasts exposed to hyperoxia and in adults with chronic obstructive lung disease [37, 38]. In our study, the longitudinal postnatal trajectory of PAI in infants with BPD indicated initial higher levels and a greater decline the first postnatal weeks. APP has previously been shown as downregulated in BPD infants [39], similar to the protein pattern in our study. Li et al. also showed a negative correlation between APP expression and neutrophil count [39]. To the best of our knowledge, CD84 and SDC4 have not been implicated in BPD pathology. CD84 is involved in immune regulation and T-cell interactions. SDC4 is involved in cell adhesion and migration and has been related to sepsis-associated lung injury [40]. In this study, differences in longitudinal trajectories of CD84, APP, and LAP TGF-β1by BPD status appeared to persist longer in infants with low BW z-score. This finding could indicate that growth and vascular processes are related to BPD development in small for gestational age infants, in line with growth restriction as a known risk factor for smaller lung volumes and cardiovascular morbidities [41, 42]. APOM and PCSK9 have been related to cardiovascular disease and lung function in adults but, to our knowledge, not in BPD [43].

Besides exposures directly related to inflammation and sepsis, several other interventions of neonatal care, such as mechanical ventilation, oxygen supplementation, and blood transfusions, are likely to contribute to immune signalling and inflammatory processes in the lungs [4]. Zhai et al. showed in a study of children with cystic fibrosis that the combination of elevated inflammatory biomarkers and lower levels of SCGB1A1 was associated with worse lung function [44]. The balance between immune-regulatory and pro-inflammatory proteins may be critical also in BPD pathogenesis. We speculate that a first exposure, such as chorioamnionitis or fetal inflammation, reduces protective mediators (for example secretoglobins), thereby sensitising the lungs to a second insult, including sepsis or oxygen supplementation. This hypothesis warrants confirmation in experimental models and larger clinical studies. Future studies should further investigate the interplay between early susceptibility and postnatal exposures, particularly in relation to immune regulation.

Infants born at GA 26–27 weeks have a lower risk of BPD compared to infants born at GA 23–24 weeks [6]. Nevertheless, some more mature infants develop severe BPD, underscoring the value of understanding their pathophysiology. In our analysis, differences in SCF trajectories were only evident among the most mature infants. SCF is expressed in endothelial progenitor cells in foetal lung tissue known to stimulate angiogenesis and alveolar formation [32, 45], and reduced expression of SCF has been reported in lungs of infants with BPD [45].

Several of the proteins in our study have previously been associated with BPD, supporting robustness of our main results. Unlike prior predominantly cross-sectional work, our study includes extremely preterm infants with dense longitudinal sampling and detailed clinical phenotyping, enabling us to characterise early postnatal dynamics in protein profiles. Nonetheless, given the observational design, we cannot distinguish whether the identified proteins reflect BPD-specific biology or are markers of disease severity and treatment exposure, such as mechanical ventilation or corticosteroids. Despite the relatively large size for a cohort of extremely preterm infants, numbers were insufficient for in-depth subgroup and exposure-specific analyses. While some findings are consistent with previous reports, confirmation in larger cohorts is warranted, particularly for GA- and BW-dependent subgroup analyses, and all results require validation in independent datasets. We employed targeted proteomics using panels selected for relevance in neonatal disease and development. This approach may increase the likelihood of validating previous findings but could miss relevant signals detected by untargeted proteomics approaches. Proteomic data were only available for a few of the deceased infants in this study, and since BPD was assessed at 36 weeks PMA, all infants who died before this time point were excluded from the analysis. This limits the generalizability of our findings to infants surviving to the time of BPD assessment. As this cohort was derived from a randomized controlled trial in extremely preterm infants, caution is also warranted when generalizing the findings to the broader preterm population.

Our results show that proteins related to BPD development span diverse biological processes, underscoring to the complexity of preterm lung development. Consistent with previous work, the data suggest that increased endothelial activation, leukocyte adhesion, transmigration, and pulmonary inflammation, in combination with disturbed protective mechanisms, contribute to lung injury and impaired alveolarization in BPD. Diverging protein trajectories further indicate that the dynamics between immune pathways and protective factors are important. We also observe subgroup differences, with growth restricted and more mature infants showing features influencing the disease process. These results need confirmation in larger cohorts but could support more targeted risk stratification and prevention.

## Conclusion

Our serum proteomic findings reinforce established BPD mechanisms and suggest additional explanatory paths and protein biomarker candidates. Several proteins may reflect vulnerability to specific exposures. Mitigating inflammation is likely of great importance for optimising lung development in preterm infants.

## Supplementary Information

Additional file 1: Table S1. Perinatal and neonatal characteristics in infants without BPD and with any grade of BPD by gestational age tertiles. Figure S1. Longitudinal protein trajectories by graded BPD outcome. Figure S2. Clinical variables and variability in protein levels the first four postnatal weeks. Figure S3. Longitudinal protein patterns by BPD stratified by GA tertiles. Figure S4. Longitudinal protein patterns by BPD stratified by BW z-score tertiles.

Additional file 2: Table S2. 108 proteins with different longitudinal protein trajectory between infants with and without BPD based on the likelihood ratio test. Table S3. 35 proteins with significant interaction term between BPD and postnatal age in any of the spline functions in the adjusted mixed model.

## Data Availability

The datasets generated and/or analysed during the current study are not publicly available due to ethical permits and The General Data Protection Regulation (GDPR) Regulation (EU) 2016/679 on the protection of natural persons with regard to the processing of personal data and on the free movement of such data law regulates the availability of personal data, but deidentified data are available from the corresponding author on reasonable request.

## Abbreviations

APP: Amyloid precursor protein
APOM: Apolipoprotein M
AA: Arachidonic acid
AUC: Area under the curve
BW: Birth weight
BST-1: Bone marrow stromal cell antigen 1
BPD: Bronchopulmonary dysplasia
CRP: C-reactive protein
DHA: Docosahexaenoic acid
FDR: False discovery rate
FCRL1: Fc receptor-like protein 1
GA: Gestational age
HSP27: Heat shock protein 27
Hb: Hemoglobin
IL: Interleukin
ICAM-1: Intercellular adhesion molecule 1
LAP TGF-beta1: Latency-associated peptide transforming growth factor beta-1
CD84: Leukocyte differentiation antigen CD84
MCP-3: Monocyte chemoattractant protein-3
MCFD2: Multiple coagulation factor deficiency protein 2
NPX: Normalized protein expression
OMD: Osteomodulin
Plt: Platelet count
PMA: Postmenstrual age
PCSK9: Proprotein convertase subtilisin kexin 9
PSP-D: Pulmonary surfactant-associated protein D
PTPRS: Receptor-type tyrosine-protein phosphatase S
ROP: Retinopathy of prematurity
SCGB3A1: Secretoglobin family 3A member 1
SCGB3A2: Secretoglobin family 3A member 2
SCF: Stem cell factor
SDC4: Syndecan-4
TFPI: Tissue factor pathway inhibitor
