# Supplementary material for: Early-life serum protein profiles associated with bronchopulmonary dysplasia in extremely preterm infants
Source: Respir Res. 2026 Sep 18;27:361. doi: 10.1186/s12931-026-03920-y (PMC13587511; doi:10.1186/s12931-026-03920-y)
Supplement: Supplementary file 1 [file 12931_2026_3920_MOESM1_ESM.pdf]

Early-life serum protein profiles associated with bronchopulmonary dysplasia in extremely preterm infants

ONLINE SUPPLEMENT

Table S1. Perinatal and neonatal characteristics in infants without BPD and with any grade of BPD by gestational age tertiles.

Figure S1 Longitudinal protein trajectories by graded BPD outcome.

Figure S2 Clinical variables and variability in protein levels the first four postnatal weeks.

Figure S3 Longitudinal protein patterns by BPD stratified by GA tertiles

Figure S4 Longitudinal protein patterns by BPD stratified by BW z-score tertiles

Table S1. Perinatal and neonatal characteristics in infants without BPD and with any grade of BPD by gestational age tertiles.

	Lowest gestational age tertile		Median gestational age tertile		Highest gestational age tertile	
**Variables**	**No BPD N=13**	**BPD any grade N=47**	**No BPD N=32**	**BPD any grade N=29**	**No BPD N=33**	**BPD any grade N=22**
Gestational age (weeks)	24 (23.0; 25.0)	24 (23.0; 25.0)	26 (25.1; 26.4)	26 (25.1; 26.4)	27.1 (26.7; 27.9)	27.1 (26.6; 27.9)
Birth weight (g)	680 (470; 790)	632 (425; 850)	856 (581; 1160)	800 (455; 1010)	1010 (523; 1330)	959 (655; 1345)
Standardized birth weight (z-score)	0.2 (-0.7; 1.4)	0.1 (-2.1; 1.6)	0.3 (-1.3; 1.8)	0.2 (-2.3; 1.1)	0.1 (-2.0; 1.4)	0.2 (-1.5; 2.3)
Sex (Female)	7 (54%)	21 (45%)	14 (56%)	13 (45%)	13 (39%)	7 (32%)
Surfactant	11 (85%)	41 (87%)	18 (56%)	19 (66%)	18 (55%)	14 (64%)
Ventilator days, 4 weeks (days)	19 (6; 28)	25 (0; 28)	1 (0; 18)	13 (0; 28)	0 (0; 9)	3 (0; 21)
Cumulative dose postnatal steroids, 4 weeks (mg/kg)	0 (0; 28)	26 (0; 201)	0 (0; 12)	0 (0; 50)	0 (0; 10)	0 (0; 117)
Sepsis, 4 weeks	3 (23%)	6 (13%)	3 (9%)	3 (10%)	2 (6%)	6 (27%)
Sum blood transfusion, 4 weeks (mL/kg)	58 (45; 132)	69 (0; 144)	30 (0; 139)	59 (0; 120)	20 (0; 64)	29 (10; 147)
Days with parenteral nutrition, 4 weeks (days)	18 (14; 28)	19 (8; 28)	13 (4; 28)	16 (4; 28)	9 (5; 27)	11 (4; 28)
Min Hb, 4 weeks (g/L)	103 (78; 111)	99 (55; 115)	105 (40; 128)	100 (38; 121)	105 (87; 154)	104 (65; 149)
Min Plt, 4 weeks (g/L)	136 (51; 178)	120 (14; 320)	171 (36; 400)	140 (25; 275)	187 (40; 388)	160 (8; 364)
Max CRP, 4 weeks (mg/L)	26 (0.5; 94)	12 (0.5; 171)	6 (0.5; 142)	11 (0.5; 54)	2.5 (0.5; 30)	12 (0.5; 217)

BPD=bronchopulmonary dysplasia; CRP=C-reactive protein; Hb=Hemoglobin; Plt=Platelet count
Data are presented as median (minimum, maximum), number of observations (percentage).

Figure S1 Longitudinal protein trajectories by graded BPD outcome.

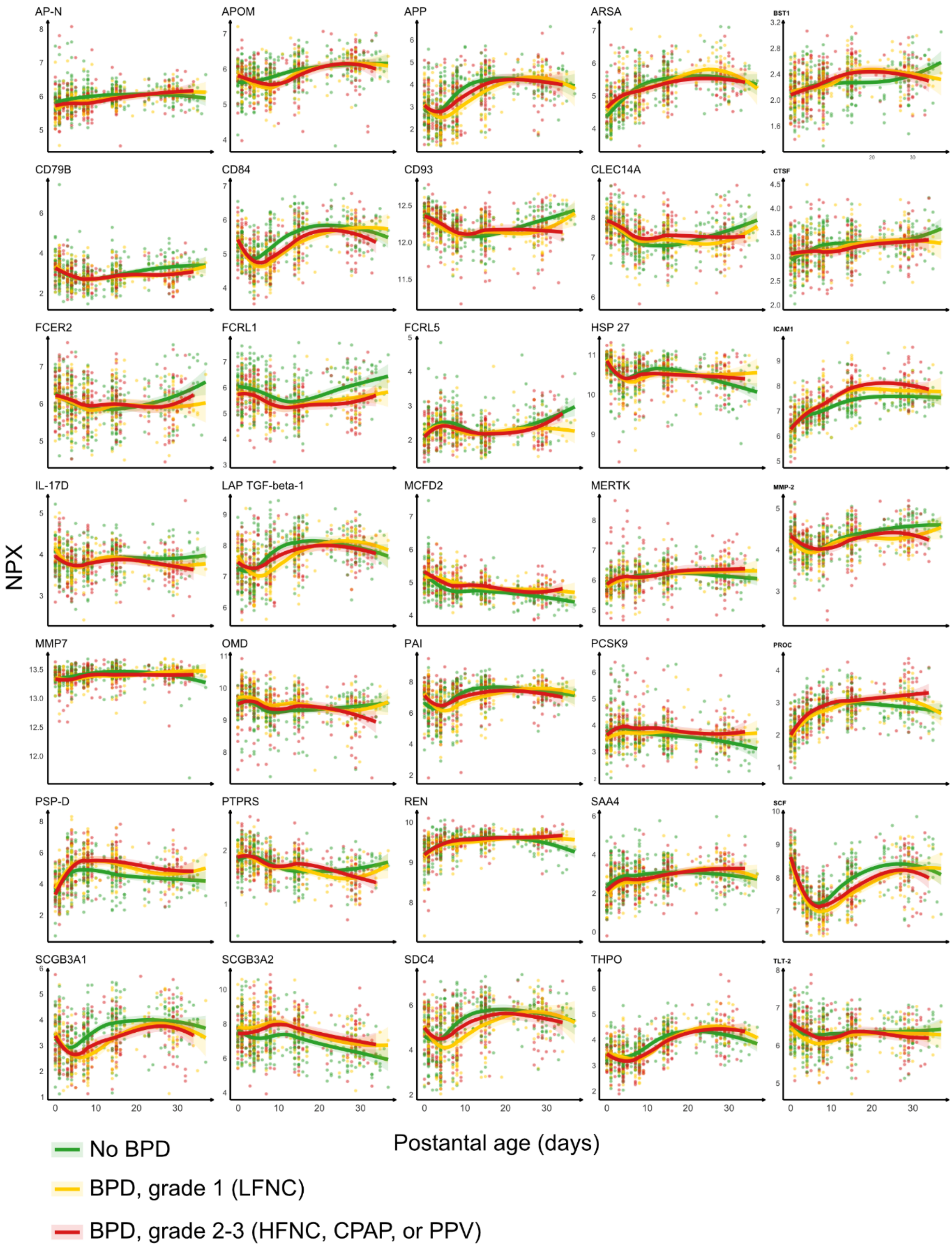

Longitudinal protein trajectories by graded BPD outcome for the 35 proteins with an FDR-significant interaction between BPD and postnatal age.

Figure S2. Clinical variables and variability in protein levels the first four postnatal weeks.

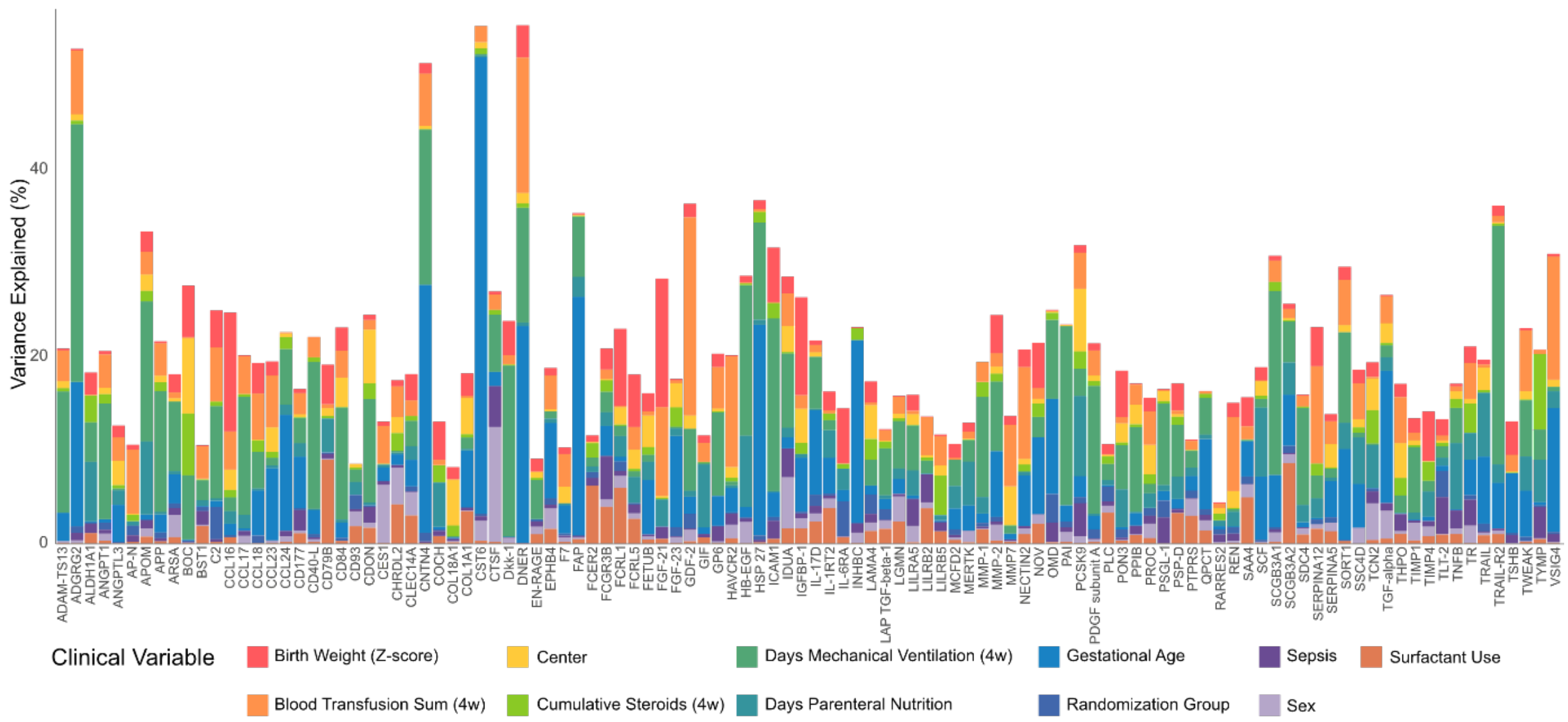

Partial R^2 from linear regression models for each explanatory variable in a multivariable model including GA, center, randomization group, BW z-score, sex, use of surfactant, sepsis, days of mechanical ventilation, days of parenteral nutrition, cumulative dose of steroids, and sum of blood transfusion the first four postnatal weeks with protein AUC as the outcome variable.

Figure S3. Longitudinal protein patterns by BPD stratified by GA tertiles

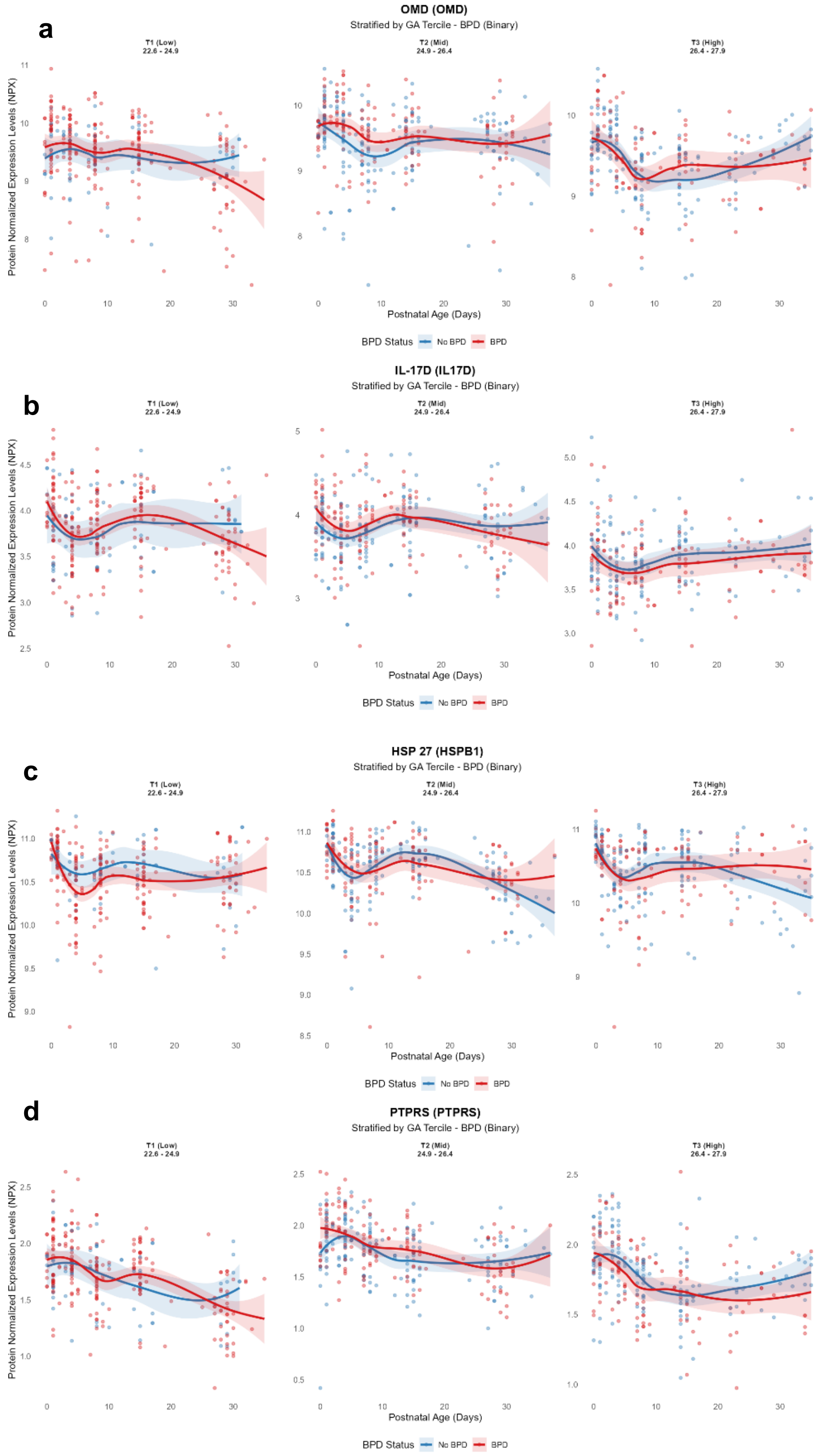

Protein levels the first five postnatal weeks by BPD status in GA tertiles for a) OMD; b) IL-17D; c) HSPB1; d) PTPRS

p-value for BPD×gestational age tertile interaction <0.05 in a mixed model of protein pattern over time including BPD status, randomization group, study center, GA, and BPD × time interaction with a natural cubic spline function.

Figure S4 Longitudinal protein patterns by BPD stratified by BW z-score tertiles

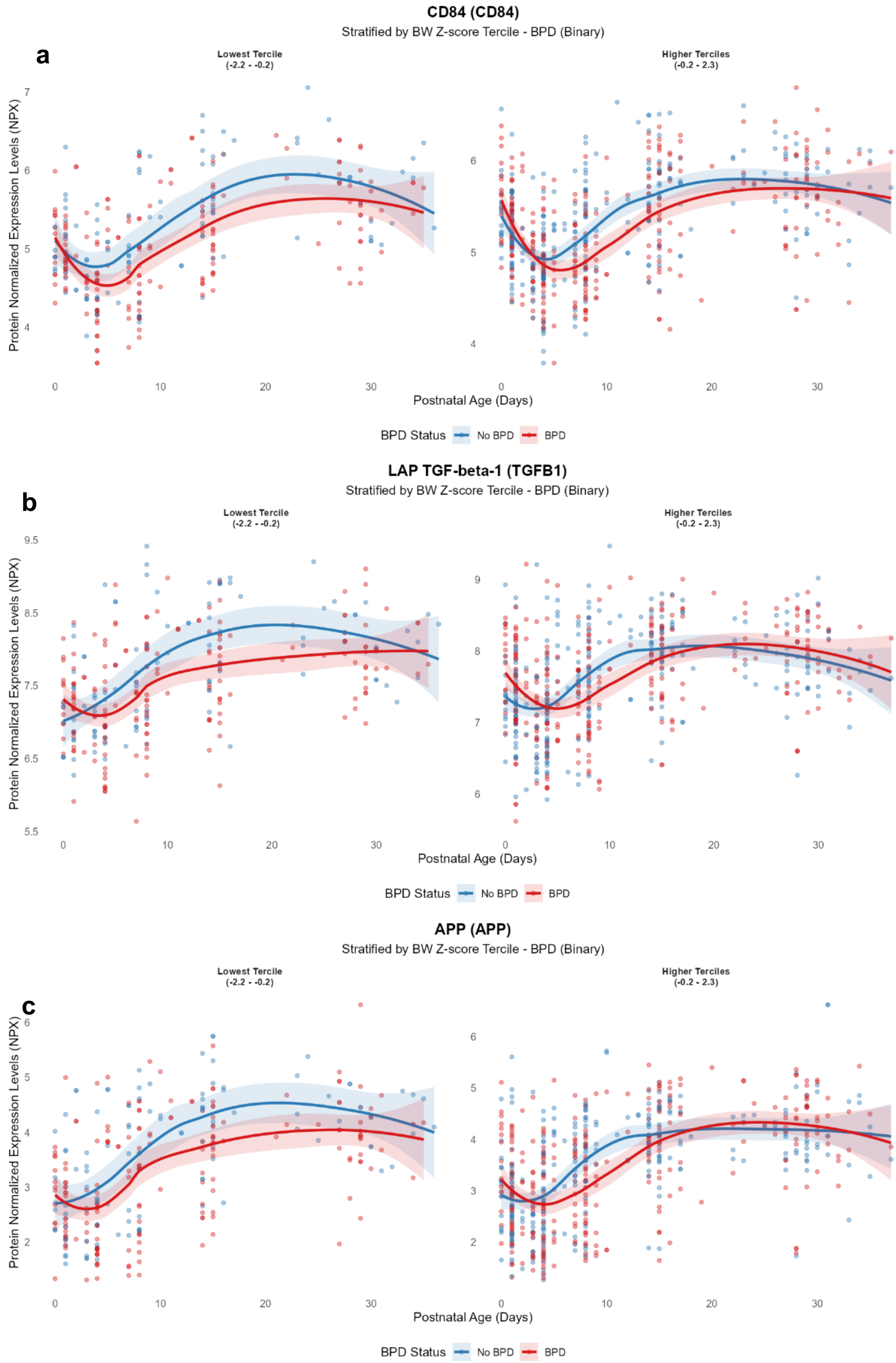

Protein levels the first five postnatal weeks by BPD status in infants with the lowest BW tertile and the two higher BW tertiles for a) CD84; b) LAP TGF-beta-1; c) APP

p-value for BPD×birth weight tertile interaction <0.05 in a mixed model of protein pattern over time including BPD status, randomization group, study center, GA, BW, and BPD × time interaction with a natural cubic spline function.
